# Wide Variation of *Aedes albopictus* Genotypes First Introduced into Canary Islands Assessed by rDNA Internal Transcribed Spacer Region and mtDNA *cox*1 Sequencing and Cloning

**DOI:** 10.3390/tropicalmed10020035

**Published:** 2025-01-27

**Authors:** Alejandra De Elías-Escribano, Irene Serafín-Pérez, Patricio Artigas, Carolina Fernández-Serafín, Sara Rodríguez-Camacho, Beatriz Yanes-Manrique, Víctor González-Alonso, Santiago Mas-Coma, Jacob Lorenzo-Morales, María Dolores Bargues

**Affiliations:** 1 Departamento de Parasitología, Facultad de Farmacia, Universidad de Valencia, Av. Vicente Andrés Estellés s/n, 46100 Valencia, Spain; alejandra.elias@uv.es (A.D.E.-E.); s.mas.coma@uv.es (S.M.-C.); 2 CIBER de Enfermedades Infecciosas (CIBERINFEC), Instituto de Salud Carlos III, C/Monforte de Lemos 3-5. Pabellón 11. Planta 0, 28029 Madrid, Spain; ireneser@ull.edu.es (I.S.-P.); cfserafin@ull.edu.es (C.F.-S.); jmlorenz@ull.edu.es (J.L.-M.); 3 Instituto Universitario de Enfermedades Tropicales y Salud Pública de Canarias (IUETSPC), Universidad de La Laguna (ULL), 38203 San Cristóbal de La Laguna, Spain; sararodcam@funccet.org (S.R.-C.); byanesma@ull.edu.es (B.Y.-M.); alu0101037394@ull.edu.es (V.G.-A.); 4 Departamento de Obstetricia y Ginecología, Pediatría, Medicina Preventiva y Salud Pública, Toxicología, Medicina Legal y Forense y Parasitología, Universidad de La Laguna (ULL), 38023 San Cristóbal de La Laguna, Spain

**Keywords:** *Aedes albopictus*, first report on Canary Islands, rDNA/mtDNA sequencing and cloning, molecular haplotyping

## Abstract

*Aedes albopictus*, one of the most rapidly spreading invasive mosquito species, has expanded from Asia to establish populations on every continent except Antarctica, showcasing exceptional adaptability, particularly in island environments. This study provides the first molecular characterization of *Ae. albopictus* in the Canary Islands, Spain. Genotyping was conducted using rDNA 5.8S-ITS2 and mtDNA *cox*1 sequencing, with haplotype analysis and phylogenetic network assessment. Among 49 sequences, 28 distinct 5.8S-ITS2 haplotypes were identified, with individual specimens containing 5 to 17 haplotypes (mean, 10.6). Most haplotypes (26/28; 92.85%) were unique to Tenerife, while only two (7.14%) were shared with other regions. H1 was the most frequent haplotype, shared with Valencia and China, while H2, a short-length haplotype, was shared with Mallorca. For *cox*1, only two haplotypes were detected: *cox*1-H1, reported in Europe, China, and Brazil, and a novel haplotype, *cox*1-H28. This genetic diversity suggests the species’ potential capacity to colonize new environments. The findings provide a foundation for further research in the Canary Islands and globally, particularly in regions with high tourism and arbovirus risks, emphasizing the importance of ongoing surveillance and genetic studies to understand the dynamics and public health impacts of invasive mosquito species.

## 1. Introduction

Vector-borne diseases account for more than 17% of infectious diseases worldwide, among which mosquito-borne diseases are the most prevalent [1]. *Aedes (Ae.) albopictus*, commonly known as the Asian tiger mosquito, is one of the fastest spreading invasive mosquito species and a competent vector for at least 26 arboviruses, including dengue, Zika, chikungunya, and yellow fever [2]. Originally from Southeast Asia, *Ae. albopictus* has successfully colonized every continent except Antarctica over the past 50 years [3]. Its global spread has been attributed to passive transport, such as the trade in used tires [4] and ornamental plants (lucky bamboo), among other goods [5,6]. Human transport networks were also involved in the spread of the Asian tiger mosquito [7]. Its invasive nature, combined with its competence as a vector, poses a significant public health challenge in many countries. This challenge is further exacerbated in oceanic islands, which, due to their isolation, small size, and high human impact, are especially vulnerable to biological invasions and diseases [8].

The presence of *Ae. albopictus* has been documented on several islands in the Atlantic Ocean, such as those in the Gulf of Guinea [9] and São Tomé and Príncipe [8]; in the Pacific Ocean, such as Hawaii [10]; and in the Indian Ocean, such as Mayotte [11]. Additionally, it has been reported in various European islands in the Mediterranean Sea, including Cyprus [12], Corsica [13], and the Balearic Islands [14,15].

In Spain, the first detection of *Ae. albopictus* was in Barcelona in 2004 [16]. It afterwards spread and established throughout the Mediterranean region including the Balearic Islands and some inland and northern areas of Spain [17]. In the Balearic Islands, its presence was first recorded in Mallorca in 2012 [14], followed by Ibiza (2014) [18] and Menorca (2016) [19]. Its spread in the Balearics has been strongly linked to tourist complexes, which were identified as key expansion indicators [15].

The Canary Islands (Spain), located in the Atlantic Ocean and only 95 km from the northwest coast of Africa, is an archipelago at constant risk of the entry of invasive aedine species due to their extensive flight and ship links with regions where these species are present. Therefore, in 2013, the archipelago joined a national initiative entitled “Entomological surveillance in airports and ports against imported vectors of exotic infectious diseases and monitoring of potential autochthonous vectors”. This project, co-funded by the Spanish Ministry of Health and the Canary Islands Health Service, involves the deployment of traps at points of entry (PoEs), such as seaports, airports, and greenhouses, throughout the archipelago, in accordance with European guidelines established by the European Centre for Disease Prevention and Control [20].

As a result of this surveillance, *Aedes aegypti*, another competent vector and significant invasive species, has been sporadically detected on the islands of Fuerteventura, La Palma, and Tenerife prior to 2022. These introductions have sometimes been associated with trade or movement of plants from areas where the vector is present. However, the current intense cruise ship traffic between the Canary Islands and other nearby archipelagos, such as Madeira where the dengue outbreak occurred in 2012 [21] and Cape Verde, has also led to the entry of *Ae. aegypti* via these vessels.

In September 2023, the first notification of the detection of *Ae. albopictus* in Tenerife was made by the Dirección General de Salud Pública (DGSP) del Gobierno de Canarias (https://www3.gobiernodecanarias.org/noticias/sanidad-activa-el-protocolo-ante-deteccion-de-un-ejemplar-de-mosquito-aedes-albopictus-en-una-vivienda-en-tenerife/, accessed on 26 September 2023). This first and early detection took place thanks to entomological surveillance, demonstrating the effectiveness of the monitoring initiative.

Accurate surveillance techniques require a detailed knowledge of the genetic diversity of *Ae. albopictus*. Following this first detection, we provide a molecular characterization by means of ribosomal DNA (rDNA) internal transcribed spacer region and mitochondrial DNA (mtDNA) cytochrome c oxidase subunit 1 (*cox*1) to describe the genotype variability of the first specimens of *Ae. albopictus* introduced into the Canary Islands.

## 2. Materials and Methods

### 2.1. Sample Collection

**Figure 1:**
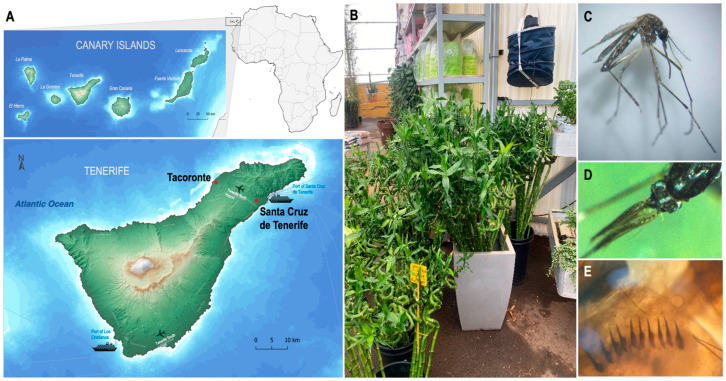
(**A**) Map of the Canary Islands archipelago and the island of Tenerife, showing the distribution of the first locations where *Ae. albopictus* were collected: Tacoronte and Santa Cruz de Tenerife. (**B**) Photograph of the surroundings of the Bg-Sentinel where the adult individuals were captured, located in the sales shed of the greenhouse. (**C**,**D**) Stereo-microscope photographs of two adults captured. (**E**) Light microscope image with a 40x lens of the larva found in the greenhouse. The scales of the eighth segment of the abdomen ending in a single central spine characteristic of *Ae. albopictus* species can be seen.

The first detection of *Ae. albopictus* occurred in a greenhouse in Tacoronte (Tenerife) (Figure 1A). A gravid female (Figure 1C) was discovered on 1 September 2023 during the examination of a Bg-Sentinel trap installed for routine entomological monitoring on the island, located in the building used to sell ornamental plants (28°31′01.47″ N, 16°24′31.60″ W). When the IUETSPC (Instituto Universitario de Enfermedades Tropicales y Salud Publica de Canarias) Medical Entomology Laboratory staff went to the greenhouse that same day to make an initial analysis of the situation, they found numerous buckets of water filled with lucky bamboo sticks (*Dracaena sanderiana*) next to the trap (Figure 1B). During the bucket inspection, an L3 stage larva was captured from the water, mounted on dimethylhydantoin-formaldehyde medium, and miscroscopically identified as *Ae. albopictus* [22] (Figure 1E).

The second detection occurred in a residential area of Santa Cruz de Tenerife (Tenerife) (Figure 1A). The presence of conspicuous bites, as well as the presence of mosquitoes morphologically compatible with an invasive aedine species, was reported by email to vectores.scs@gobiernodecanarias.org on 25 September 2023. Three male mosquitoes (Figure 1D) were caught in a Bg-Sentinel trap placed in the house (28°27′49.39″ N, 16°16′44.86″ W) where the first bites were reported.

The specimens were captured as part of the passive entomological surveillance entomological control system which it has been in action in the Canary Islands for 12 years. Sentinel traps are located in garden centers, airports and harbors, and other sensitive locations and checked weekly. The specimens collected and included in the study were the first ones detected and hence the importance to include their molecular identification.

After morphological identification [23,24], specimens of *Ae. albopictus* from both detection events were frozen at −20 °C and then fixed in absolute ethanol for sample transport and subsequent molecular analysis. The reason for a reduced sample size is justified by the fact that these are the first detected specimens. After that, surveillance included more traps in the areas of the detection which were checked weekly as well as fumigation and cleaning of the areas.

### 2.2. Molecular Study

Genomic DNA was extracted from 2–3 legs and the thorax of each one of the four adult mosquitoes (1 female from Tacoronte and 3 males from Santa Cruz de Tenerife, Tenerife, Canary Islands, Spain) using the InstaGene™ Matrix Kit (Bio-Rad Laboratories^®^, Hercules, CA, USA) according to the manufacturer’s instructions.

Two molecular markers, second internal transcribed spacer (ITS-2) of the rDNA and *cox*1 of the mtDNA, were used for specimen haplotyping. Each molecular marker was PCR-amplified independently for each sample and each PCR product was sequenced for haplotype characterization. The complete rDNA ITS-2 was amplified using primers and PCR conditions previously described [15,25,26]. Two sets of primers were used to amplify the nearly complete *cox*1 gene [27]. Sequencing was performed on both strands by the dideoxy chain termination method with Taq dye-terminator chemistry kit on an Applied Biosystems 3730xl DNA Analyser (Applied Biosystems, Foster City, CA, USA) using the same amplification PCR primers.

Cloning procedures were applied to the PCR products when double peaks were observed at many positions in the electropherograms of the ITS-2 sequences obtained by direct sequencing. The ITS-2 amplification product from each sample was cloned using the pGEM-T Easy Vector System I (Promega, Madison, WI, USA) and introduced into *Escherichia coli* DH5α competent cells. Between nine and 30 distinct white colonies were PCR amplified and sequenced individually for each cloned sample. To minimize potential cloning biases, we employed the Hot-Start version of Taq DNA polymerase (Takara Bio Europe, Saint-Germain-en-Laye, France) along with PCR primers and optimized PCR conditions as previously described [15,25,26]. The DNA sequences obtained contain almost half the length of the 5.8S gene, followed by the complete ITS-2 sequence, followed by ~10 nucleotides of the initial 5′ region of the 28S gene. DNA sequencing of the clones was performed as described above.

### 2.3. Sequence Analyses

Electropherograms were thoroughly inspected using FinchTV v. 1.5 (Geospiza, Inc., Seattle, WA, USA). Forward and reverse sequences were edited and assembled with Sequencher v. 5.4.6 (Gene Codes Co., Ann Arbor, MI, USA) and aligned using ClustalW in MEGA X v.10.2.6 software [28]. All changes, including transitions (ts), transversions (tv), and insertions/deletions (indels), were considered as character states in MEGA X. Sequences were collapsed into haplotypes via the ALTER web server [29], with gaps counted as differences. Homologies were determined using the BLAST program from the National Center for Biotechnology Information website (http://www.ncbi.nlm.nih.gov/BLAST, accessed on 23 October 2023).

DnaSP v.5.10.01 [30] was used to evaluate the number of haplotypes (H), haplotype diversity (Hd), nucleotide diversity expressed as the average number of nucleotide differences between two sequences by site (π), average number of nucleotide differences between sequences (k), and number of polymorphisms and insertions/deletions (S). Deviations from selective neutrality were analyzed using the statistics Fu’s Fs and Tajima’s D of the neutrality test by means of the DnaSP v.6 software.

### 2.4. Phylogenetic Networks

A haplotype network was generated to depict the relationships among *Ae. albopictus* rDNA haplotypes with Network 10.2.0.0. software (http://www.fluxus-engineering.com/, accessed on 11 December 2023). The network was constructed with the 5.8S-ITS2 rDNA sequences obtained, and others retrieved from GenBank (>490 bp long and 100% homology), using the median-joining (MJ) network algorithm, with default parameters (equal character weight = 10, transitions/transversions weight = 1:1, epsilon = 0, frequency > 1, and connection cost as a criterion). Hypothetical median vectors were added to the network for the shortest connection between the datasets. The reliability of the connections between haplotypes were calculated with 1000 bootstrap replicates using MEGA X v.10.2.6 software.

## 3. Results

### 3.1. rDNA ITS-2 Sequence Analysis

The complete rDNA ITS-2 sequence, along with an 80 bp fragment of the 5.8S gene, was successfully obtained from three specimens through repeated cloning and sequencing attempts. The 49 sequences obtained were grouped into 28 different haplotypes (H1 to H28) of the 5.8S-ITS-2 rDNA, considering both single nucleotide differences and indels for haplotype identification. These 28 haplotypes showed pronounced differences in length, varying between 429 and 488 bp (mean 480.2 bp) with an average GC content of 55.8%. Their alignment was 491 base pairs (bp) long and included 439 conserved positions and 52 variable sites, including 29 parsimony-informative and 23 singleton sites. The shortest sequence (429 bp) of all these haplotypes was detected in one of the clones of *Ae. albopictus* from Santa Cruz de Tenerife, generating a long region of consecutive gapped positions in the alignment with all the other sequences. This large indel region, consisting of 57 consecutive deletions, was located between positions 253 and 310 of the 5.8S-ITS2 alignment.

The large number of haplotypes found within a same individual is noteworthy, ranging from 5 to 17 haplotypes (average 10.6) in specimens from both geographical locations analyzed: 11–17 haplotypes (mean 14) in Santa Cruz; five haplotypes in Tacoronte (Table 1). None of these 28 haplotypes showed 100% identity and 100% coverage after a BLAST search except haplotypes H1 and H2 (the only one presenting a 57-nucleotide deletion), which turned out to be identical to the sequences of: *Ae. albopictus* isolate AEAE09 from Valencia, Spain (GenBank MW281941) and isolate DF02 from China (OR907176); and isolate AEAE53 from Mallorca (MW281992), Spain, respectively (Table 1).

When comparing the sequences of these 28 haplotypes, the pairwise 5.8S-ITS2 evolutionary divergence matrix obtained with MEGA X v.10.2.6 software shows that the total number of absolute differences (gaps not included) in pairwise comparisons ranged between 1 and 27 (average 8.99). The highest values were obtained when comparing haplotypes from Santa Cruz.

When comparing the 49 sequences of the three specimens representing these 28 haplotypes, the pairwise 5.8S-ITS2 distance matrix allowed us to analyze the overall mean distance at intra-specimen and inter-specimen level, showing that the total number of differences (ts + tv) ranged between 5.20–11.69 (average, 8.51) and 7.45–10.55 (8.80), respectively. The highest values were obtained when comparing intraindividual sequences and inter-individual sequences from Santa Cruz.

### 3.2. Haplotype Network

**Figure 2:**
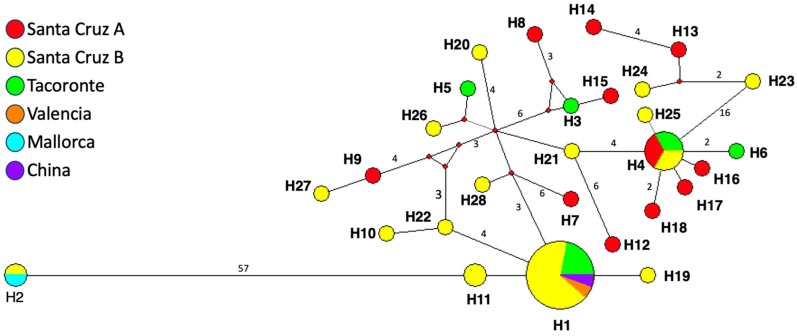
Phylogenetic network based on ITS-2 sequences of *Ae. Albopictus* haplotypes detected in Tenerife, Canary Islands, Spain, and identical sequences from GenBank. Small red-filled circles represents intermediate haplotypes not present in the sample. Mutational steps between haplotypes are represented by line length. More than one mutational step is represented by numbers. Circles are proportional to the number of samples represented for each haplotype. Colors in the legend correspond with those represented by the haplotype network.

The median joining haplotype network allows to distinguish the evolutionary branches among the 28 haplotypes of *Ae. albopictus* (Figure 2). The overall haplotype diversity (Hd) was 0.9954, the nucleotide diversity (π) was 0.01833, the average nucleotide difference number (K) was 7.552, and the number of segregating sites (S) was 40. Bootstrap support for connections between the 28 haplotypes ranged from 73% to 100%, indicating results to be highly statistically significant.

The haplotype H1 was the most abundant and shared by 18 sequences obtained from: Tacoronte (one specimen, four clones); Santa Cruz de Tenerife (specimen A, 12 clones), Valencia, Spain, isolate AEAE09 (GenBank MW281941), and China, isolate DF02 (OR907176). The second most abundant was H4, shared only by the two new localities for *Ae. albopictus* in Tenerife and obtained in six clonal sequences of specimens from Tacoronte and Santa Cruz de Tenerife.

The haplotype H2 shared between Santa Cruz de Tenerife (specimen B) and Mallorca, Spain, appears as the most distant haplotype in the network, due to its peculiar short length (429 bp, due to their long deletion region in the ITS-2).

### 3.3. mtDNA cox1 Sequence Analysis

*Cox*1 sequencing provided two 1421 bp-long haplotypes, *cox*1-H1 and *cox*1-H28, of an average 70.13% AT content, differing from each other in only one variable position located at position 27 (C or T) of their alignment. The resulting COX1 protein was 473 aa long, and yielded only one haplotype for all four samples analyzed.

The *cox*1-H1 haplotype was detected in the three male mosquitoes found in Santa Cruz de Tenerife. This haplotype was previously reported in the Spanish localities of Valencia, Barcelona, and Mallorca Island (MW279068) and in other European countries, including Albania (KX383930), Greece (KX383932), France (MW279068), Italy (KX383929), and Portugal (MN513352). It was also reported in China (KC690898) and Brazil (MK575475). The *cox*1-H28 haplotype, detected in the female mosquito analyzed from Tacoronte, proved to be a new haplotype.

## 4. Discussion

The Asian tiger mosquito is a highly invasive species and it is a competent vector for several arboviruses [8,31], enabling local transmission of these pathogens in the invaded areas. Therefore, tracking their rapid global spread is essential [3]. This is the first study on the genetic variability from the earliest *Ae. albopictus* invaders detected in the Canary Islands based on the combined use of rDNA/mtDNA markers.

The rDNA ITS-2 has proven to be a useful marker for analyzing *Ae. albopictus* spread, providing insights at the intra-individual, intra-population, and inter-population levels [15,32,33]. We describe 28 different 5.8S-ITS2 haplotypes for the two insular locations of *Ae. albopictus* in Tenerife, 26 (92.85%) of which are new for this species, while only two (7.14%) (H1 and H2) have been detected in other Spanish localities (Valencia and Mallorca) [15]. Although our current data do not allow us to infer the origin of the first *Ae. albopictus* detected in the Canary Islands, the discovery in Tenerife of two haplotypes present in Valencia and Mallorca suggests a possible introduction from mainland Spain, with which the Canary Islands have strong cultural and commercial ties.

The haplotype H4 shared by the specimens from Tacoronte and Santa Cruz de Tenerife further supports a common source of introduction. Moreover, these two localities are situated near Tenerife North Airport, which has several daily direct flights to mainland Spain and Balearic Islands, and near one of the main ports of the island (Figure 1A), potentially facilitating introduction.

Interestingly, the H1 haplotype found in Tenerife and previously detected in Valencia [15] has also been reported in China [27], supporting an Asian origin for *Ae. albopictus* populations introduced into Europe [3].

The presence of the rare haplotype H2, detected in one clone sample from Tacoronte and characterized by its short ITS-2 sequence and a large region of consecutive deletions, coincides with the two, three and six clone sequences from Mallorca, Perpignan, and Manaus, respectively [15], or the haplotype Aa45 (KY703659) from St. Denis in Reunion Island [32]. Curiously, two of these localities concern islands, like Tenerife, receiving a large number of tourists and a significant exchange of people and goods with other countries.

The high variability detected in 5.8S-ITS2 furnishes a high number of haplotypes within the same mosquito individual (5–17 haplotypes, mean 10.6, in Canary Islands), in agreement with similar reports from southwestern Europe (2–7, 4.7) and Brazil (1–6, 3.3) [15]. The number of 5.8S-ITS2 haplotypes is higher than the previously reported mean of 2 haplotypes per individual [32].

The information obtained from 5.8S-ITS2 was complemented by the almost complete sequence of *cox*1, a usual marker used for mosquito species, although it provides less information due to its conserved characteristics in *Aedes* populations [15,32,33,34]. In the present study, only two haplotypes differing in only one polymorphic position were detected, *cox*1-H1 and *cox*1-H28, highlighting the low level of polymorphisms detected, which agrees with previous studies [8,15]. The *cox*1-H1 haplotype is highly represented in specimens from other localities in Spain, such as Valencia and Mallorca [15]. This reinforces the possible introduction in Tenerife due to the intense relationships and exchanges that the archipelago has with other Spanish localities. For the *cox*1-H28 haplotype, no matches were found with previously reported sequences. Although it is true that most studies analyze only a short sequence of this gene [8,35], there is an increasing tendency to perform complete or almost complete sequencing [3,15,32], as in our study. This lack of standardization in molecular methods makes it difficult to compare results.

*Ae. albopictus* has shown a remarkable ability to effectively adapt and establish to island environments [8,10,11]. A prominent example is the Balearic Islands in Spain, where the Asian tiger mosquito has spread rapidly since its introduction, colonizing several islands in a short period of time [14,18,19]. The first local dengue outbreak was recently described in Ibiza (Balearic Islands), where *Ae. albopictus* has been present since 2014 [36]. In February 2023, German health authorities reported two dengue cases and four possible cases in people who had traveled to Ibiza in late summer 2022. This outbreak was attributed to the introduction of the virus by travelers returning from dengue-endemic areas. This event underscores the potential of this vector to transmit arboviruses in geographical areas where it has recently established and highlights the vulnerability of islands with high tourist traffic.

The Canary Islands share similar characteristics with the Balearic Islands, such as a significant influx of tourists and climatic conditions appropriate for the establishment and spread of *Ae. albopictus*. These factors emphasize the importance of proactive surveillance and the crucial role of national initiatives, such as those that facilitated the early detection of these invasive mosquitoes in the present study.

## 5. Conclusions

This study reports the first detection of *Ae. albopictus* in the Canary Islands (Tenerife) and its genetic characterization using a combination of ribosomal and mitochondrial markers. The genetic analysis revealed a remarkable number of 5.8S-ITS2 haplotypes among the first specimens collected in Tenerife.

The genetic characterization of the first *Ae. albopictus* in the Canary Islands serves as a starting point for future studies both in the archipelago and other regions, especially touristic islands at risk for arboviruses with high sanitary impact. These findings highlight the importance of ongoing surveillance and genetic studies to better understand the dynamics of invasive mosquito species and their potential impact on public health.

## Figures and Tables

**Table 1:** Number of rDNA ITS-2 haplotypes found in the cloned specimens of *Ae. albopictus* from Tenerife, Canary Islands, Spain and their BLAST homologies obtained with a 100% identity and 100% coverage.

Specimen Code/Sex	Locality Tenerife	Clones	ITS-2 rDNA Haplotypes	BLAST (100%) Identity	Species/Isolate	GenBank Acc. Number	Country, Locality
Ae-Tc/female	Tacoronte	9	5	1	*Ae. albopictus*/AEAE09 *Ae. albopictus*/DF02	MW281941 OR907176	Spain, Valencia China
Ae-SCa/male	Santa Cruz	12	11	-	-	-	-
Ae-SCb/male	Santa Cruz	28	17	1	*Ae. albopictus*/AEAE53	MW281992	Spain, Palma de Mallorca

## Data Availability

The original data presented in the study are available in the GenBank database (http://www.ncbi.nlmnih.gov, accessed on 24 October 2024) under the accession numbers PQ499584–PQ499611 and PQ499046–PQ499047.
